# Anti-tumour effects of lanreotide for pancreatic and intestinal neuroendocrine tumours: the CLARINET open-label extension study

**DOI:** 10.1530/ERC-15-0490

**Published:** 2016-03

**Authors:** Martyn E Caplin, Marianne Pavel, Jarosław B Ćwikła, Alexandria T Phan, Markus Raderer, Eva Sedláčková, Guillaume Cadiot, Edward M Wolin, Jaume Capdevila, Lucy Wall, Guido Rindi, Alison Langley, Séverine Martinez, Edda Gomez-Panzani, Philippe Ruszniewski

**Affiliations:** 1 Royal Free Hospital, London, UK; 2 Charité – University Medicine Berlin, Berlin, Germany; 3 University of Warmia and Mazury, Olsztyn, Poland; 4 University of Texas MD Anderson Cancer Center, Houston, Texas, USA; 5 University Hospital, Vienna, Austria; 6 Department of Oncology of the First Faculty of Medicine and General Teaching Hospital, Prague, Czech Republic; 7 Robert-Debré Hospital, Reims, France; 8 Markey Cancer Center, University of Kentucky, Lexington, Kentucky, USA; 9 Vall d'Hebron University Hospital, Barcelona, Spain; 10 Western General Hospital, Edinburgh, UK; 11 Università Cattolica del Sacro Cuore, Rome, Italy; 12 Ipsen, Les Ulis, France; 13 Ipsen, Basking Ridge, New Jersey, USA; 14 Beaujon Hospital, Clichy, France; 15 Paris Diderot University, Paris, France

**Keywords:** neuroendocrine tumours, lanreotide Autogel, anti-tumour effects, open-label extension

## Abstract

In the CLARINET study, lanreotide Autogel (depot in USA) significantly prolonged progression-free survival (PFS) in patients with metastatic pancreatic/intestinal neuroendocrine tumours (NETs). We report long-term safety and additional efficacy data from the open-label extension (OLE). Patients with metastatic grade 1/2 (Ki-67 ≤10%) non-functioning NET and documented baseline tumour-progression status received lanreotide Autogel 120 mg (*n*=101) or placebo (*n*=103) for 96 weeks or until death/progressive disease (PD) in CLARINET study. Patients with stable disease (SD) at core study end (lanreotide/placebo) or PD (placebo only) continued or switched to lanreotide in the OLE. In total, 88 patients (previously: lanreotide, *n*=41; placebo, *n*=47) participated: 38% had pancreatic, 39% midgut and 23% other/unknown primary tumours. Patients continuing lanreotide reported fewer adverse events (AEs) (all and treatment-related) during OLE than core study. Placebo-to-lanreotide switch patients reported similar AE rates in OLE and core studies, except more diarrhoea was considered treatment-related in OLE (overall diarrhoea unchanged). Median lanreotide PFS (core study randomisation to PD in core/OLE; *n*=101) was 32.8 months (95% CI: 30.9, 68.0). A sensitivity analysis, addressing potential selection bias by assuming that patients with SD on lanreotide in the core study and not entering the OLE (*n*=13) had PD 24 weeks after last core assessment, found median PFS remaining consistent: 30.8 months (95% CI: 30.0, 31.3). Median time to further PD after placebo-to-lanreotide switch (*n*=32) was 14.0 months (10.1; not reached). This OLE study suggests long-term treatment with lanreotide Autogel 120 mg maintained favourable safety/tolerability. CLARINET OLE data also provide new evidence of lanreotide anti-tumour benefits in indolent and progressive pancreatic/intestinal NETs.

## Introduction

CLARINET was a landmark 96-week study that confirmed anti-tumour effects for the long-acting somatostatin analogue lanreotide Autogel (depot in the USA) 120 mg. Lanreotide prolonged progression-free survival (PFS) over placebo in patients with metastatic grade 1 or 2 (proliferation index, Ki-67, up to 10%) somatostatin-receptor positive pancreatic, intestinal or of unknown primary origin neuroendocrine tumours (NETs) with prior stable disease (SD), irrespective of hepatic tumour volume (Caplin *et al*. 2014). Consequently, lanreotide Autogel/depot 120 mg is now indicated in both the US and EU as an anti-tumour treatment for pancreatic and intestinal NET (Ipsen 2014, 2015). Furthermore, this novel evidence for lanreotide has prompted re-evaluation of guidelines for first-line medical therapy of metastatic gastroenteropancreatic NETs (NCCN 2014, Pavel *et al*. 2016).

Here, we present data from the pre-planned interim analysis of the ongoing single-arm (lanreotide Autogel 120 mg) open-label extension (OLE) to the core study. The primary objective of the OLE study was to investigate the long-term safety of lanreotide in patients with pancreatic and intestinal NETs. Safety/tolerability of lanreotide was investigated not only in patients receiving lanreotide in both the core and OLE studies but also in patients switching from placebo (core study) to lanreotide (OLE). Another objective was to further examine anti-tumour efficacy. In the core study, median PFS for placebo was 18.0 months, but had not been reached with lanreotide after 96 weeks. This analysis provided an opportunity to estimate the median PFS for lanreotide (in continued lanreotide patients) and to determine the median time to subsequent progression in placebo-to-lanreotide patients who had progressive disease (PD) in the core study.

## Patients and methods

### Patients

Inclusion and exclusion criteria for the core study have been published (Caplin *et al*. 2014) but are summarised here to characterise the CLARINET OLE population.

The core study included adult patients with sporadic, well- or moderately differentiated NETs that had proliferation index (Ki-67) of up to 10% and were non-functioning, except for gastrinomas adequately controlled with proton pump inhibitors for ≥4 months. Primary tumours were located in the pancreas, midgut or hindgut, or were of other/unknown origin. Patients were also required to have an unresectable locally advanced tumour or metastatic disease (or had declined surgery), target lesion(s) classified as at least grade 2 (Krenning scale) within the previous 6 months and a World Health Organization (WHO) performance score of ≤2. Core study exclusion criteria comprised treatment with interferon, chemoembolisation or chemotherapy within 6 months before study entry, radionuclide therapy at any time or a somatostatin analogue at any time (unless received over 6 months previously for <15 days); major surgery related to the NET within the previous 3 months; multiple endocrine neoplasia; or previous cancer (except *in situ* cervical or uterine carcinoma or basal-cell skin carcinoma or other cancers treated with curative intent and disease-free for >5 years).

Patients were eligible for the OLE study if attending a participating study centre and if they had SD (according to response evaluation criteria in solid tumours (RECIST) 1.0) at the end of the 96-week core study (while receiving lanreotide or placebo) or had centrally assessed PD during the core study while receiving placebo (Supplementary Figure S1, see section on supplementary data given at the end of this article). Patients' WHO performance score had to remain at ≤2 at the start of the OLE study.

Patients could be withdrawn from the OLE study if local assessments indicated tumour progression, or for safety reasons. Patients could withdraw therapy at their own request at any time.

### Trial design and interventions

The CLARINET OLE is a single-arm, non-randomised, multicentre study conducted in 24 centres across ten countries (in Europe, India and USA). Patients were enrolled within 4 weeks of their last core study visit and received lanreotide Autogel 120 mg by deep s.c. injection every 28 days. The study started in February 2009 and will continue in each country until registration for tumour control. This pre-planned interim analysis was undertaken at the time of last patient's last visit in the core study (data cut-off: March 2013).

Patients provided written informed consent. Study protocol, amendments (amendments occurring after the start of the study are summarised in the Supplementary Materials and methods, see section on supplementary data given at the end of this article) and consent form were approved by independent ethics committees in each country; the Declaration of Helsinki, Good Clinical Practice guidelines and all local regulatory requirements were adhered to. The study was registered at ClinicalTrials.gov (NCT00842348) and EudraCT (2008-004019-36).

### Assessments and endpoints

The OLE study assessments (multiphase CT or dynamic contrast-enhanced MRI scans) were scheduled for week 1 (OLE study baseline) and every 24 weeks, as well as at the time of early withdrawal (if applicable) or at any time if PD was suspected. Adverse events (AEs) were recorded throughout the study on 4-weekly treatment visits. Other safety assessments included physical examination, assessment of vital signs and clinical laboratory tests (all visits), and electrocardiography and ultrasonography of the gallbladder conducted at baseline, weeks 48 and 96, or early withdrawal visit. Scans were assessed locally for the signs of PD according to RECIST 1.0, compared to core study baseline scan or a subsequent scan indicating a nadir in core or OLE studies (patients with SD in core study), or OLE study baseline or a subsequent scan indicating a nadir in OLE study (patients with PD in core study).

The primary objective of the OLE study was to investigate the long-term safety of lanreotide Autogel 120 mg in patients with pancreatic and intestinal NETs. The secondary objective was to further investigate its efficacy. This interim analysis provides an opportunity to estimate median PFS for lanreotide in patients originally randomised to and continuing to receive lanreotide, both overall (main efficacy endpoint) and for pre-specified as well as clinically relevant subgroups (see Supplementary Materials and methods). The interim analysis also facilitates estimation of the time to subsequent PD in patients switching to open-label lanreotide after PD while receiving placebo in the core study (additional efficacy endpoint). Insufficient numbers precluded evaluation of subsequent PD in subgroups.

### Statistical analysis

Descriptive statistics were calculated for safety data from the safety population (all patients who received at least one dose of lanreotide in the OLE study). Descriptive rather than inferential statistics were performed because this was a non-comparative OLE study, and there were some inherent differences between patients who switched to active drug from placebo or continued on active drug, notably in SD/PD status at the start of this study. Safety analyses were described for groups according to the sequence of treatment received during the core and OLE studies (lanreotide in both studies or placebo in core, followed by lanreotide in OLE study).

PFS (main efficacy end point) and the time to subsequent PD with lanreotide (additional efficacy end point) were described using Kaplan–Meier curves with events defined as deaths and PD (centrally assessed during core and locally assessed during OLE study, all using RECIST 1.0); all other outcomes were censored as per Food and Drugs Administration guidance (FDA 2007). Two sensitivity analyses were performed (see the Supplementary Materials and methods for details and for information on the populations used for efficacy analyses). Efficacy data were presented in months approximated to 4 weeks.

Statistical analyses were performed using SAS software version 9.3 (SAS Institute, Inc.).

## Results

### Patient disposition and characteristics

Of patients from the core study (*n*=138) eligible for the OLE study, 79 patients had SD at the core study end (53 on lanreotide and 26 on placebo) and 59 had PD while receiving placebo during the core study. In total, 52 (37.7%) patients who were eligible did not enrol, mostly (37/52, 71.2%) because their study centres did not participate in the OLE study. Reasons for non-enrolment were not documented for the remaining 15 patients.

**Figure 1:**
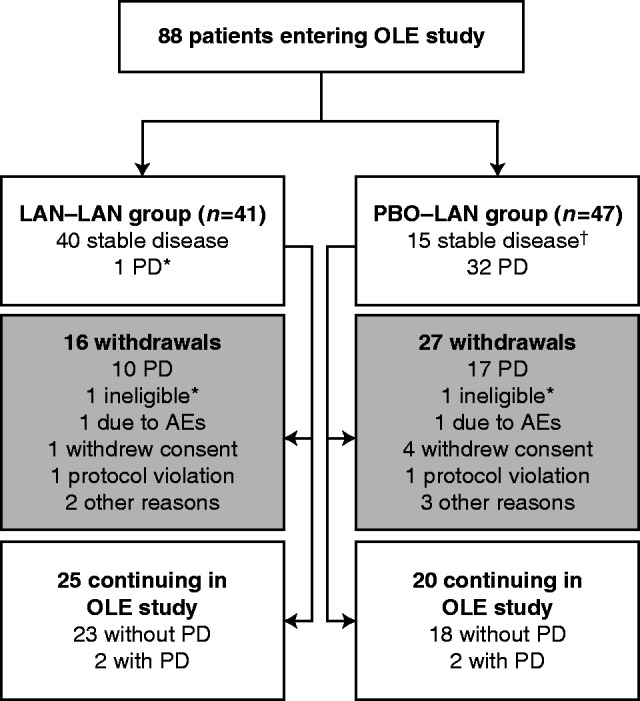
Flow of patients through the OLE study. These numbers refer to status at the time of the OLE interim analysis. For further information, see Supplementary Figure S1. *One patient was enrolled by investigator prior to confirmation of centrally assessed PD (patient then withdrawn when confirmation received). ^†^One patient was withdrawn from the core study for a reason other than centrally assessed PD but was then enrolled in the OLE. AE, adverse event; LAN, lanreotide autogel 120 mg; OLE, open-label extension; PBO, placebo; PD, progressive disease.

Of 88 patients enrolled in the OLE study (among whom two were ineligible, Fig. 1 and Supplementary Figure S1), 41 continued on lanreotide and 47 patients switched from placebo to lanreotide. At the time of the pre-planned interim analysis, none of the 88 participants had died.

Compared with the groups with SD on lanreotide or placebo at the end of the core study, the group with PD while receiving placebo contained a greater proportion with worse WHO performance status (scores of 1 or 2) at baseline in the OLE study and a greater proportion with a NET originating in the pancreas (Table 1).

### Treatment exposure

The medians (ranges) for combined lanreotide exposure in the core and OLE studies were 40.0 (26.0–74.3) months in the continued lanreotide group, 18.1 (1.0–49.9) months in the placebo-to-lanreotide group with SD at the end of the core study and 13.0 months (2.0–52.0) in the placebo-to-lanreotide group with PD during the core study.

### Safety/tolerability

In the continued lanreotide group, incidences of overall and individual AEs (whether considered related to treatment or irrespective of causality) were generally lower for the OLE study compared with the core study (Table 2). This difference was particularly marked for diarrhoea and abdominal pain (Supplementary Tables S1 and S2, see section on supplementary data given at the end of this article).

In the placebo-to-lanreotide group, the overall incidence of AEs, whether treatment-related or not, was similar across the core and OLE studies (Table 2). This pattern was reflected across most individual AEs, except for diarrhoea. Whereas the incidence of diarrhoea irrespective of causality was similar between studies (most common AEs occurring in ≥10% of patients shown in Supplementary Table S1), the incidence considered to be treatment-related was higher in the OLE study (most common treatment-related AEs occurring in ≥5% of patients shown in Supplementary Table S2). Incidences of severe and serious AEs were similar between groups and across studies. Only one patient was withdrawn due to a treatment-related AE (placebo-to-lanreotide group: non-serious tumour haemorrhage associated with serious tumour necrosis in the liver, the patient recovered with sequelae following hospitalisation and appropriate treatment).

The gallbladder echography was conducted on at least two occasions during the core and OLE studies combined in 29/41 (71%) patients in the continued lanreotide group, and 34/47 (72%) patients in the placebo-to-lanreotide group. Among these patients, new lithiasis and/or new sludge were detected in four patients each in the placebo-to-lanreotide group and six patients each in the continued lanreotide group.

No clinically significant trends were observed in other safety assessments.

### Efficacy

**Figure 2:**
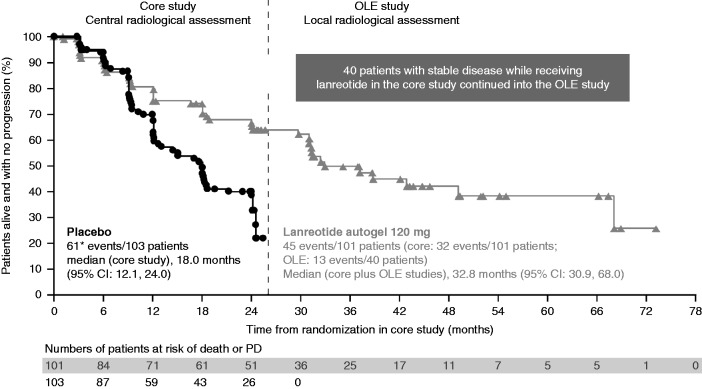
PFS on lanreotide in the core and OLE studies, at the time of the pre-planned interim analysis, and on placebo in the core study (ITT population). Data in months are approximated based on 4 weeks per month. Median PFS was not reached for patients receiving lanreotide Autogel 120 mg during the 24-month core study (vs 18.0 months for patients receiving placebo). Core study data are for all patients randomly allocated to double-blind treatment (lanreotide or placebo); the OLE study data are only for patients originally randomly allocated to lanreotide in the core study who then continued into the OLE study. *Previously reported as 60 events (Caplin *et al*. 2014) because one patient was erroneously reported as having centrally assessed SD at the time of core study database lock; this has been revised in the OLE study analysis. ITT, intention-to-treat population; OLE, open-label extension; PD, progressive disease.

Median PFS was not reached for lanreotide in the 96-week core study (Caplin *et al*. 2014). With data from the OLE study at the time of the pre-planned interim analysis appended to data from the core study, median PFS in patients receiving lanreotide Autogel 120 mg was estimated to be 32.8 months (95% CI: 30.9, 68.0) (Fig. 2). In total, 23 patients from this continued lanreotide group are ongoing in the OLE study without PD (Fig. 1).

Supportive analysis (based on the per-protocol population) provided the same estimate of median PFS for lanreotide as the main (intention-to-treat (ITT)) analysis: 32.9 months (95% CI: 30.9, 68.0). In the *a priori* sensitivity analysis, data from 15 patients who were withdrawn from the core study due to investigator assessment of PD (despite central assessment of SD) were added as events. Compared with the main analysis, median (95% CI) PFS for lanreotide in the sensitivity analysis was similar (31.3 months (24.0, 49.0)). In the *post hoc* sensitivity analysis, data from 25 patients (13 receiving lanreotide) with SD at the end of the core study but who did not enter the OLE study were added to the analysis, and all were assumed to have had PD at the first OLE study assessment. Median PFS for lanreotide in this sensitivity analysis was consistent with the main analysis: 30.8 months (95% CI: 30.0, 31.3).

**Figure 3:**
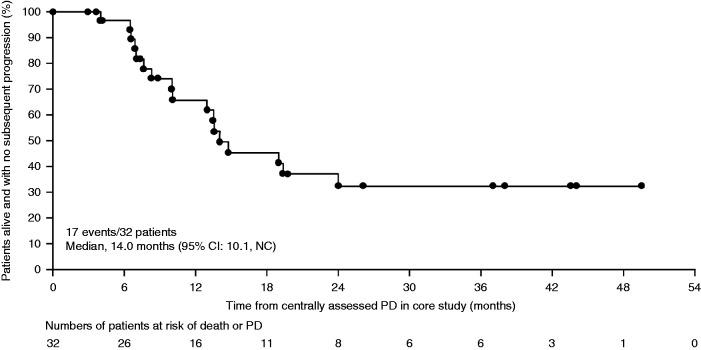
Time to death or subsequent PD, at the time of the pre-planned interim analysis, in patients with PD on placebo in the core study who switched to lanreotide in the OLE study (subset of the ITT population). Data in months are approximated based on 4 weeks per month. Data are from patients originally randomised to placebo in the core study, who have experienced PD in the core study and then switched to lanreotide Autogel 120 mg in the OLE study. NC, not calculable. ITT, intention-to-treat population; OLE, open-label extension; PD, progressive disease.

For patients who had PD (*n*=32) while receiving placebo in the core study and continued into the OLE study (receiving open-label lanreotide), 17 had subsequent PD during the OLE; a median time from first PD in the core study with placebo to subsequent PD in the OLE study with lanreotide was 14.0 months (95% CI: 10.1, not reached) (Fig. 3). Nine patients are continuing to receive lanreotide treatment in the OLE study without subsequent PD (reasons for discontinuation are shown in Fig. 1 and Supplementary Figure S1). For patients without PD on placebo after completing the full 96 weeks of the core study (*n*=15), three patients experienced PD with lanreotide Autogel 120 mg in the OLE study (one of three who withdrew for PD did not have PD; one patient was ongoing with PD at time of interim analysis). Nine patients were continuing lanreotide treatment without PD at the time of interim analysis.

For continued lanreotide patients, PFS results in the core plus OLE studies across subgroups based on tumour origin, grade, hepatic tumour load, PD and previous therapy status at core study baseline and region were generally consistent with the main analysis (Supplementary Table S3, see section on supplementary data given at the end of this article). Numbers of placebo-to-lanreotide patients were insufficient to assess time to subsequent PD across subgroups after switching to lanreotide.

## Discussion

The CLARINET OLE study further investigates the safety and efficacy of lanreotide in patients with metastatic pancreatic and intestinal NETs. The long-term safety and tolerability profile of lanreotide Autogel 120 mg was favourable during a median treatment duration of 40 months (continued lanreotide group; range: 26–74 months). Among patients who received lanreotide in the core and OLE studies, the pattern of most AEs suggests amelioration with increasing duration of treatment exposure, particularly for diarrhoea (whether treatment-related or not). In the group switching from placebo in the core study to lanreotide in the OLE study, the pattern of AEs between studies shows generally comparable rates, including treatment-related AEs except for diarrhoea, which was higher in the OLE study. The latter finding was not unexpected, as diarrhoea is a common transient side effect after the initiation of somatostatin analogue therapy.

The safety and tolerability profile of lanreotide is well-evidenced from its use as a treatment for symptoms of carcinoid syndrome in clinical studies (Ruszniewski *et al*. 2004, Bajetta *et al*. 2006) and many years of clinical practice in numerous countries worldwide (Khan *et al*. 2011, Palazzo *et al*. 2013). This experience is consistent with the favourable safety data observed in the CLARINET core study and this OLE study. Indeed, the safety/tolerability profile shown here further supports the use of this agent early in the treatment pathway for NETs and over the long term. The incidence and nature of treatment-related AEs among patients switching from placebo to lanreotide in the OLE either align broadly with or compare favourably to those in other studies (Ruszniewski *et al*. 2004, Bajetta *et al*. 2006). Furthermore, a lower incidence of AEs, in particular diarrhoea and abdominal pain, occurred in the group continuing lanreotide treatment in the OLE study.

Overall, the efficacy data are consistent with continued anti-tumour effects. Key findings include the 32.8-month estimate for median PFS in patients originally randomised to lanreotide in the core study, which contrasts with the 18.0-month median PFS for placebo reported previously (Caplin *et al*. 2014). The OLE study data provided an estimated 14.0-month time to progression with lanreotide in patients with PD on placebo in the core study; this information is clinically important, with the caveat that it is based on data available for only 32 of 59 potentially eligible patients.

The CLARINET core study is, to date, the most comprehensive and robust study of the anti-tumour effects of a somatostatin analogue in patients with metastatic NETs. Lanreotide significantly prolonged PFS in a large population with advanced grade 1 or 2 (Ki-67 up to 10%) pancreatic and intestinal NETs and indolent disease (96% SD according to RECIST), irrespective of hepatic tumour load; median PFS for lanreotide was not reached (Caplin *et al*. 2014). The OLE efficacy data extend the duration of lanreotide use among the CLARINET patient population and have allowed the median PFS to be estimated. These observations accord well with limited data on anti-tumour treatment with lanreotide available before CLARINET from observational studies. A single-centre UK study reported a 5-year PFS rate of 59% with lanreotide for 76 patients with mostly low-grade midgut NETs (Khan *et al*. 2011). A multicentre study from France of lanreotide treatment found median PFS of 29 months in a cohort of 68 patients with metastatic NETs (foregut 28%/midgut 59%; 54% liver burden ≤25%) (Palazzo *et al*. 2013).

The CLARINET OLE study additionally provides evidence that lanreotide has an anti-tumour effect in patients with PD. The 14.0-month estimated PFS in the population of patients with progressive pancreatic and intestinal NETs is similar to the 12.9 months estimated in a phase II uncontrolled study involving 30 patients with progressive NETs (lung 13%/gastrointestinal 47%/pancreatic 27%) receiving lanreotide for ≤92 weeks (Martin-Richard *et al*. 2013). Time-to-progression in two patients with midgut NETs treated with octreotide after progressing on placebo in the PROMID study was 14.0 and 16.0 months (Rinke 2012) (31% of the OLE PD subgroup had midgut NETs). Although a direct comparison is not feasible, the CLARINET data are broadly consistent with PFS reported in phase III studies of other targeted agents in patients with progressive pancreatic NETs (11.0 months for everolimus in the RADIANT-3 study (Yao *et al*. 2011), 11.4 months for sunitinib (Raymond *et al*. 2012)), although only 53% of the PD subgroup in the CLARINET OLE study had pancreatic NETs. Together then, the core and OLE data advance our understanding of the anti-tumour effects of lanreotide, supporting its positive benefit/risk profile as one of the recommended first-line therapy options for metastatic pancreatic and intestinal NETs either for prevention or for inhibition of tumour growth (NCCN 2014).

Undoubtedly, extensions of large randomised controlled trials such as CLARINET are important; however, the OLE study is not without limitations. Firstly, not all eligible core study patients continued to be followed into the OLE study, principally owing to non-participation of some core study centres; nonetheless, a degree of selection bias may have occurred. However, as noted, this issue was addressed in a *post hoc* sensitivity analysis that included all patients with SD in the core study who did not continue into the OLE study and designated them as having PD at the first OLE assessment. Reassuringly, median PFS from this sensitivity analysis accorded well with the main analysis. Secondly, the OLE study was not specifically designed to measure efficacy, and in contrast to the core study, it was open-label, lacked a control group and based PFS estimates on locally rather than centrally assessed PD. Nevertheless, the estimate of median PFS for lanreotide was based on the ITT population from the randomised controlled core study and, in large part, on events confirmed centrally during the core study.

In summary, the CLARINET OLE study demonstrated that the long-term treatment with lanreotide Autogel 120 mg was well-tolerated, with no indication of new safety concerns. Notwithstanding study limitations, the CLARINET OLE data suggest that lanreotide Autogel 120 mg has anti-tumour effects in patients with PD. Additionally, the CLARINET core and OLE studies together provide evidence for long-term PFS benefits of lanreotide Autogel 120 mg in patients with indolent pancreatic and intestinal NETs.

## Supplementary data

This is linked to the online version of the paper at http://dx.doi.org/10.1530/ERC-15-0490.

## Figures and Tables

**Table 1:** Demographic and disease characteristics for participants in the OLE study according to the treatment sequence they received and their PD status during the core study (safety population)

	**LAN–LAN group** (*n*=41)	**PBO–LAN group**	
No PD during core study (*n*=15)	PD during core study (*n*=32)
Men, *n* (%)	18 (43.9)	6 (40.0)	19 (59.4)
Age, mean (s.d.) in years^a^	64.9 (10.9)	59.5 (12.0)	62.1 (9.3)
Time since diagnosis, mean (s.d.) in months	36.5 (58.8)	34.7 (45.0)	45.2 (47.5)
WHO performance status score, *n* (%)^a^			
0 – normal activity	35 (85.4)	13 (86.7)	21 (65.6)
1 – restricted activity	6 (14.6)	2 (13.3)	10 (31.3)
2 – in bed ≤50% of the time	0	0	1 (3.1)
Prior NET treatment, *n* (%)	5 (12.2)	4 (26.7)	5 (15.6)
NET origin, *n* (%)			
Pancreas	11 (26.8)	5 (33.3)	17 (53.1)
Midgut	17 (41.5)	7 (46.7)	10 (31.3)
Hindgut	5 (12.2)	1 (6.7)	1 (3.1)
Other/unknown	8 (19.5)	2 (13.3)	4 (12.5)
Tumour progression at, *n* (%)			
Core study baseline	0	1 (6.7)	3 (9.4)
OLE study baseline	1 (2.4)^b^	0	32 (100)
Tumour grade, *n* (%)			
G1 (Ki-67 0–2%)	30 (73.2)	12 (80.0)	20 (62.5)
G2 (Ki-67 3–10%)^c^	11 (26.8)	3 (20.0)	12 (37.5)
Hepatic tumour load, *n* (%)			
0%	9 (22.0)	2 (13.3)	10 (31.3)
>0–10%	19 (46.3)	10 (66.7)	9 (28.1)
>10–25%	1 (2.4)	3 (20.0)	4 (12.5)
>25–50%	10 (24.4)	0	5 (15.6)
>50%	2 (4.9)	0	4 (12.5)

Data are from assessments at core study baseline or ^a^OLE study baseline and are for patients receiving lanreotide Autogel 120 mg in both CLARINET core and OLE studies (LAN–LAN group), and patients receiving placebo in the core study and crossing over to lanreotide in the OLE study (PBO–LAN group).

b Patient enrolled by the investigator before communication of the results of the central assessment (PD) in the core study; patient withdrawn from the OLE study upon receipt of the assessment results.

c Ki-67 thresholds as per WHO 2010 classification with values >2 to ≤10% assigned to tumour grade 2. LAN, lanreotide Autogel 120 mg; NET, neuroendocrine tumour; OLE, open-label extension; PBO, placebo; PD, progressive disease; SD, standard deviation; WHO, World Health Organization.

**Table 2:** Incidence of AEs in the core and OLE studies among participants of the OLE study according to the treatment sequence (safety population)

	**LAN–LAN group** (*n*=41)			**PBO–LAN group** (*n*=47)	
Core study	OLE study	Both studies (pooled)	Core study	OLE study
Any patients with an AE	38 (92.7)	27 (65.9)	39 (95.1)	44 (93.6)	38 (80.9)
Treatment-related	22 (53.7)	11 (26.8)	25 (61.0)	13 (27.7)	19 (40.4)
Severe	11 (26.8)	10 (24.4)	16 (39.0)	11 (23.4)	11 (23.4)
Moderate	17 (41.5)	11 (26.8)	16 (39.0)	26 (55.3)	20 (42.6)
Mild	9 (22.0)	6 (14.6)	6 (14.6)	7 (14.9)	7 (14.9)
Missing	1 (2.4)	0	1 (2.4)	0	0
Any patients with serious AEs	10 (24.4)	9 (22.0)	15 (36.6)	10 (21.3)	10 (21.3)
Treatment-related	3 (7.3)	1 (2.4)^a^	3 (7.3)	1 (2.1)	1 (2.1)^a^
Withdrawals due to AEs	NA^b^	2 (4.9)^c^	2 (4.9)	NA	1 (2.1)^c^
Treatment-related	NA^b^	0	0	NA	1 (2.1)
Most common individual AEs^d^					
Diarrhoea	14 (34.1)	4 (9.8)	15 (36.6)	15 (31.9)	15 (31.9)
Abdominal pain	12 (29.3)	3 (7.3)	13 (31.7)	7 (14.9)	6 (12.8)
Constipation	8 (19.5)	2 (4.9)	9 (22.0)	5 (10.6)	1 (2.1)
Nausea	7 (17.1)	2 (4.9)	8 (19.5)	5 (10.6)	4 (8.5)
Dizziness	7 (17.1)	2 (4.9)	7 (17.1)	1 (2.1)	2 (4.3)
Cholelithiasis	6 (14.6)	4 (9.8)	9 (22.0)	5 (10.6)	2 (4.3)
Headache	6 (14.6)	2 (4.9)	8 (19.5)	6 (12.8)	4 (8.5)
Vomiting	6 (14.6)	2 (4.9)	8 (19.5)	4 (8.5)	5 (10.6)
Hypertension	6 (14.6)	2 (4.9)	7 (17.1)	3 (6.4)	5 (10.6)

Data are number (%) of patients with an AE while receiving lanreotide Autogel 120 mg or placebo. AEs were defined according to the Medical Dictionary for Regulatory Activities (MedDRA) version 16.0. LAN–LAN group, patients receiving lanreotide Autogel in core study as well as the OLE study; PBO–LAN, patients receiving placebo in the core study before crossing over to lanreotide in the OLE study. AE, adverse event; OLE, open-label extension.

a One patient experienced cholelithiasis (LAN–LAN group) and one experienced tumour necrosis (PBO–LAN group).

b NA, not applicable (no patients withdrawn from the core study were entered into the OLE study).

c At the time of the pre-planned interim analysis of the OLE study, two withdrawals had been reported due to AEs in the LAN–LAN group: ileus was not considered related to treatment, and tumour pain was subsequently confirmed not to be the reason for withdrawal (withdrawal due to protocol violation); one patient in the PBO–LAN group withdrew due to tumour necrosis (also reported as a serious AE) and tumour haemorrhage, which were reported to be treatment related.

d Based on MedDRA preferred terms, full list of AEs occurring in ≥10% patients are listed in Supplementary Table S1, and treatment-related AEs occurring in ≥5% patients are listed in Supplementary Table S2.
